# News from Societies

**Published:** 1956-04

**Authors:** 


### News from Societies

### CORNWALL CLINICAL SOCIETY

1955

Friday, 4th November: Clinical Evening. Camborne-Redruth Hospital.
Thursday, 24th November: Dinner and Dance. Hotel Bristol, Newquay
Thursday, 1st December: Clinical Evening. R.C.I.

1956

Friday, 6th January: Clinical Ebening. Camborne-Redruth Hospital.
Thursday, 2nd February: Films: "*Valvotomy*", "*The Conjoined Twins of Kano*". R.C.I.
Friday, 2nd March; Dr. F. E. Camps, "*Death at Rillington Place*". R.C.I.
Friday, 6th April: Clinical Evening. Camborne-Redruth Hospital.
Thursday, 3rd May; Clinical Evening. R.C.I.
Friday, 1st June: Clinical Evening. Chest Hospital, Tehidy.
Friday, 6th July: Cases and Demonstrations by Consultant Staff. Camborne-Redruth Hospital.
Friday, 5th October: Presidential Address and A.G.M. Camborne-Redruth Hospital.

1. Refreshments will be served from 8.0 to 8.30 p.m.
2. The meeting will start at 8.30 p.m. and finish not later than 10.30 p.m.
3. Detailed notices of individual meetings will be circulated in advance.
4. The Secretaries will be pleased to receive suggestions or clinical contributions at any time.

J. D. Hardy,
T. S. Wilson,
Hon. Secs.

### DEVON AND EXETER MEDICO-CHIRURGICAL SOCIETY

At a meeting on 29th September, 1955, Mr. H. Dendy Moore, F.R.C.S., spoke of the uses of diodone, an organic iodide known to be safe, in diagnosis and treatment. Venography with this medium can be used to show normal venous anatomy, deep varices, old thromboses and recanalizations, permanent blocks in veins, acute thromboses and particularly the state of the valves. At present, though useful for teaching and research, it is of little practical value in surgery. By percutaneous puncture of an artery and the injection of diodone, arterial blocks and aneurysms can be shown. Even the aorta may be punctured directly and safely through the back. Multiple X-ray exposures though useful are not essential. Intravascular injections of diodone enable abdominal organs to be visualized. Splenography, an easy and safe procedure, can demonstrate the results in portal hypertension of various origins and also hepatic lesions. Following aortagrams the kidneys can be visualized and by multiple X-ray exposures the passage of diodone through, and its excretion by, the kidney can be seen. Diodone can also be used to show congenital abnormalities of the kidney, the various stages of hydronephrosis, hypernephromata and pyelonephritis. The stomach can be outlined if a small amount of viscous diodone is swallowed and the site of a perforated gastric or duodenal ulcer demonstrated. Diodone is also of value in cases of ruptured bladder, of empyaema and of sinus infection.

### WESSEX RAHERE CLUB

The Spring Dinner of the club will take place at the Royal Clarence Hotel, Exeter, on Saturday, 28th April, 1956.

It is hoped that Mr. Derrick Colthart will be present as Guest of Honour.

Membership of the club is open to all Bart's men practising in the West Country. Further details will be circulated to members and to any other Bart's men who are interested and who will get in touch with the Hon. Secretary, Mr. A. Daunt Bateman, of 11 The Circus, Bath.

